# Supplementary material for: Development and validity evidence for the intraprofessional conflict exercise: An assessment tool to support collaboration
Source: PLoS One. 2023 Feb 17;18(2):e0280564. doi: 10.1371/journal.pone.0280564 (PMC9937497; doi:10.1371/journal.pone.0280564)
Supplement: S1 Fig — (DOCX) [file pone.0280564.s001.docx]

**Supporting Information Figure 1:** **The Intraprofessional Conflict Exercise Development Process**
