# Supplementary material for: Development and validity evidence for the intraprofessional conflict exercise: An assessment tool to support collaboration
Source: PLoS One. 2023 Feb 17;18(2):e0280564. doi: 10.1371/journal.pone.0280564 (PMC9937497; doi:10.1371/journal.pone.0280564)
Supplement: S1 Table — (DOCX) [file pone.0280564.s002.docx]

**Supporting Information Table 1: Example of the Intraprofessional Conflict Exercise**

**INTRAPROFESSIONAL CONFLICT EXERCISE**

Evaluator: _________________________________________________________

Student/Resident: ___________________________________________________

Level: (please check) **ٱ 3rd yr ٱ 4th yr ٱ res 1 ٱ res 2 ٱ res 3 ٱ res 4 ٱ res 5**

|  | N/A | Unacceptable | Below Expectations | Meets Expectations | Exceeds Expectations |
| --- | --- | --- | --- | --- | --- |
| Listened actively to colleague |  |  |  |  |  |
| Showed interest in the colleague as a person |  |  |  |  |  |
| Uses nonverbal communication to convey openness and supportiveness |  |  |  |  |  |
| Identifies nonverbal cues from colleague |  |  |  |  |  |
| Demonstrates empathy for their colleague |  |  |  |  |  |
| Uses “I” statements to effectively communicate their emotions and point of view |  |  |  |  |  |
| Identifies the needs or interests of the colleague |  |  |  |  |  |
| Demonstrated awareness of limitations |  |  |  |  |  |
| Describes the situation in terms of a shared observation |  |  |  |  |  |
| Admitted errors/omissions |  |  |  |  |  |
| Solicited feedback |  |  |  |  |  |
| Accepted feedback |  |  |  |  |  |
| Maintained appropriate boundaries |  |  |  |  |  |
| Maintained composure in a difficult situation |  |  |  |  |  |
| Maintained appropriate appearance |  |  |  |  |  |
| Addressed own gaps in knowledge and skills |  |  |  |  |  |
| Demonstrated respect for colleagues |  |  |  |  |  |
| Avoided derogatory language |  |  |  |  |  |
| Avoids verbal attacks on the colleague |  |  |  |  |  |
| Negotiates a mutually agreeable solution to the conflict |  |  |  |  |  |
| Verbalizes specific solutions to the conflict |  |  |  |  |  |

► Please rate this student’s/resident’s overall professional performance during THIS encounter:

**ٱ** UNacceptable  **ٱ** MET expectations **ٱ** BELow expectations **ٱ** EXCeeded expectations

► Did you observe a critical event? **ٱ no ٱ yes** (comment required)

Comments:_________________________________________________________________________________________________________________________________________________________________________________________________________________________________________________________________________________________________________________________________________________________

Evaluator’s signature: ______________________________________

Student’s/Resident’s signature: ______________________________

Date & Time: ____________________________________________
