# Supplementary material for: Development and validity evidence for the intraprofessional conflict exercise: An assessment tool to support collaboration
Source: PLoS One. 2023 Feb 17;18(2):e0280564. doi: 10.1371/journal.pone.0280564 (PMC9937497; doi:10.1371/journal.pone.0280564)
Supplement: Attachment — Submitted filename: Response to Reviewers_Bajwa_PlosOne_V3.docx [file pone.0280564.s004.docx]

December 12th, 2022

Dear Prof. Gilligan,

We would like to thank you for your further consideration of our manuscript (PONE-D-22-19898), “Development and validity evidence for the Intraprofessional Conflict Exercise: An assessment tool to support collaboration.”

In this second revision, my co-authors and I have carefully reviewed and made every effort to address each concern in this revision.

Again, we are very pleased to have the opportunity to share our findings with the health sciences community through publication in *PLOS ONE*. We thank you for considering our manuscript and await your reply.

Sincerely yours,

Nadia Bajwa, MD, MHPE, PhD

Médecin Adjointe, Pédiatrie Générale, Responsable de la Formation Post-graduée, HUG

Membre facultaire, Unité de Développement et de Recherche en Education Médicale (UDREM), UNIGE

Département de la femme, de l'enfant et de l'adolescent

6, rue Willy-Donzé - 1211  Genève 14

Tél. [+41 22 37 23082](tel:%2B41%2022%2038%2023082), +41 79 55 33705

Email: [nadia.bajwa@hcuge.ch](mailto:nadia.bajwa@hcuge.ch)

**Manuscript ID#:** PONE-D-22-1989

**Manuscript Title: Development and validity evidence for the Intraprofessional Conflict Exercise: An assessment tool to support collaboration**

Journal Requirements:

Please review your reference list to ensure that it is complete and correct. If you have cited papers that have been retracted, please include the rationale for doing so in the manuscript text, or remove these references and replace them with relevant current references. Any changes to the reference list should be mentioned in the rebuttal letter that accompanies your revised manuscript. If you need to cite a retracted article, indicate the article’s retracted status in the References list and also include a citation and full reference for the retraction notice.

-> All references have been reviewed and verified to comply with the PLOS One style.

Additional Editor Comments:

Thank you for responding to the reviewers' earlier comments. I can see that you have comprehensively done so. In the absence of a second reviewer for this revision I have reviewed the manuscript and am happy to say that pending a few minor changes I think it will soon be ready to be accepted for publication.

Please consider:
1. The information you have added on the bottom of page 6 (to address reviewer queries about the setting, transferability of findings, previous communication skills training and application in different stages of health professions education) seems to be a little clumsy in that it is all together and some of it somewhat out of place. I would suggest that most parts of this would be best placed in the limitations section in the discussion, with the exception of the reference to application in undergraduate vs postgraduate training.

->We thank the Editor for their comment. We have now reformulated the section on page 6 and have also moved the discussion on prior communication skills training to the limitations in the discussion:

*“Most applicants completed medical school at the University of Geneva where communication skills training includes but is not limited to conducting an interview with a patient, breaking bad news, and conducting handoffs. Conflict management is not specifically addressed. The use of the ICE between undergraduate and graduate medical education was appropriate given applicants’ likely previous exposure to training in communication. While the use of the ICE is limited in our study to a residency admissions process, the ICE is intended to be used in a variety of settings as part of conflict management training at any stage of the health professions education continuum.”*

Limitations:

*“In addition, participants were not questioned on their previous communication skills or conflict management training and this may have had an impact on their performance.”*

2. I am still not entirely clear on the justification for not trialing this with undergraduate students – using a workplace scenario makes sense but why not test this with students to see how it works as a teaching tool before they enter the workplace? The goals and conclusions all point to application for medical student training but the ICE has not been tested in that context.

-> We thank the editor for their valid comment. We have now added this perspective to our limitations:

“The study of the use of the ICE with various intraprofessional collaboration challenges, specifically those concerning patient care, would add to the ICE’s validity evidence. More specifically, we would like to see the application of the ICE in an undergraduate teaching setting on conflict management with the goal of preparing students for the workplace.”
Typo:
-Abstract - final sentence of methods and findings section - there is a repeat word 'in'

->We thank the editor for their careful read and have made the correction.


Reviewers' comments:

Reviewer's Responses to Questions

**Comments to the Author**

1. If the authors have adequately addressed your comments raised in a previous round of review and you feel that this manuscript is now acceptable for publication, you may indicate that here to bypass the “Comments to the Author” section, enter your conflict of interest statement in the “Confidential to Editor” section, and submit your "Accept" recommendation.

Reviewer #1: All comments have been addressed

2. Is the manuscript technically sound, and do the data support the conclusions?

The manuscript must describe a technically sound piece of scientific research with data that supports the conclusions. Experiments must have been conducted rigorously, with appropriate controls, replication, and sample sizes. The conclusions must be drawn appropriately based on the data presented.

Reviewer #1: Yes

3. Has the statistical analysis been performed appropriately and rigorously?

Reviewer #1: I Don't Know

4. Have the authors made all data underlying the findings in their manuscript fully available?

The [PLOS Data policy](http://www.plosone.org/static/policies.action#sharing) requires authors to make all data underlying the findings described in their manuscript fully available without restriction, with rare exception (please refer to the Data Availability Statement in the manuscript PDF file). The data should be provided as part of the manuscript or its supporting information, or deposited to a public repository. For example, in addition to summary statistics, the data points behind means, medians and variance measures should be available. If there are restrictions on publicly sharing data—e.g. participant privacy or use of data from a third party—those must be specified.

Reviewer #1: Yes

5. Is the manuscript presented in an intelligible fashion and written in standard English?

PLOS ONE does not copyedit accepted manuscripts, so the language in submitted articles must be clear, correct, and unambiguous. Any typographical or grammatical errors should be corrected at revision, so please note any specific errors here.

Reviewer #1: Yes

6. Review Comments to the Author

Please use the space provided to explain your answers to the questions above. You may also include additional comments for the author, including concerns about dual publication, research ethics, or publication ethics. (Please upload your review as an attachment if it exceeds 20,000 characters)

Reviewer #1: The authors have addressed all my comments. The article makes a valuable contribution to the literature addressing the complexity of and education for healthcare practice.

7. PLOS authors have the option to publish the peer review history of their article ([what does this mean?](https://journals.plos.org/plosone/s/editorial-and-peer-review-process#loc-peer-review-history)). If published, this will include your full peer review and any attached files.


If you choose “no”, your identity will remain anonymous but your review may still be made public.


**Do you want your identity to be public for this peer review?** For information about this choice, including consent withdrawal, please see our [Privacy Policy](https://www.plos.org/privacy-policy).

Reviewer #1: No
